# DEVELOPMENT OF MITOPHAGY-INDUCING SMALL MOLECULES AS THERAPEUTICS FOR AGING AND ALZHEIMER’S DISEASE

**DOI:** 10.1093/geroni/igad104.0732

**Published:** 2023-12-21

**Authors:** Julie Andersen

**Affiliations:** Buck Institute for Research on Aging, Novato, California, United States

## Abstract

Alzheimer’s disease (AD) is the leading cause of dementia in those over the age of 65. The underlying molecular basis for the disease remains elusive. Rather than simply viewing individual diseases in isolated silos, researchers have recently turned to study of the contribution of common age-related processes to age-related diseases. One such shared process is loss in mitochondrial function, normally maintained by clearance of defective mitochondria via mitophagy. Mitophagy slows with age and its pre-clinical and clinical elevation has been shown to slow various age-related conditions. An agent which has received considerable attention in this regard is the compound urolithin A (UA). Several labs, including our own, have recently reported neuroprotection in association with its administration in a variety of mouse AD models. As part of an independent small molecule screen carried out by the lab, we identified a second structurally-related compound we named ‘mitophagy inducing compound’ (MIC). Using C. elegans as an initial validation tool, we showed that both compounds significantly improve mitochondrial function, protect against neuronal cell loss in disease models, and extend lifespan of wildtype animals. These protective effects were found to be translatable to both in vitro and in vivo mammalian models. To identify potential drug targets, we next turned to Cellular Thermal Shift Assay (CETSA). Preliminary data from knockdown of identified targets suggest involvement of a mito-hormetic mechanism which acts to restore overall mitochondrial health. These studies may have important implications for understanding signaling events elicited by these compounds driving their disease-modulating effects.

